# Kinetics of cytokine profile in response to *Mycobacterium bovis* BCG and *Streptococcus pyogenes* activated cells

**DOI:** 10.1016/j.dib.2016.02.061

**Published:** 2016-03-03

**Authors:** Vivek Verma, Parveen Kumar, Rakesh Singh Dhanda, Manisha Yadav

**Affiliations:** a Dr. B.R. Ambedkar Centre for Biomedical Research (ACBR), University of Delhi, Delhi 110007, India; b Department of Translational and Regenerative Medicine, Post Graduate Institute of Medical Education & Research (PGIMER), Chandigarh 160012, India

**Keywords:** *Mycobacterium bovis* BCG, *Streptococcus pyogenes*, Cytokines, IL-8, IL-6, TNF-α

## Abstract

The infection of epithelial cells is a necessary step for *Mycobacterium bovis* BCG dissemination, but the mechanism of mycobacterial epithelial interactions is not completely understood. Similarly, *Streptococcus pyogenes* is a strictly human pathogen that favorably colonizes the skin and the pharynx. Effective cytokine secretion is essential in order to fabricate a suitable inflammatory response against an infection. In this data article, the cytokine profile in BCG and *S. pyogenes* activated THP-1 cell line in media after the acute phase of infection by ELISA is described. The interleukin-8 level was increased in response to both BCG and *S. pyogenes,* but was quite prominent after 24 h and further increased upto 72 h post infection. On the other hand, an increase in IL-6 response to *S. pyogenes* was observed while there was no response to BCG even after 48 h of infection. A low level of TNF-α was detected upon BCG and *S. pyogenes* infection.

## 1 **Specifications table**

**Table:** 

Subject area	*Biology*
More specific subject area	*Medical microbiology, Mycobacterium bovis* BCG, *Streptococcus pyogenes*
Type of data	*Graphs*
How data was acquired	*Cytokine profile*
Data format	*Analyzed*
Experimental factors	*Kinetics of cytokine profile*
Experimental features	Kinetics of cytokine profile in response to BCG and *S. pyogenes*
Data source location	*University of Delhi, New Delhi, India*
Data accessibility	*Data is with this article only*

## 2 **Value of the data**

• Data shows induction of IL-6 and IL-8 secretion by human macrophages upon *S. pyogenes* infection, which is an important pro-inflammatory response through TLRs sensitization, necessary for immune recognition and downstream immune responses.
• BCG infection alone induces IL-8 levels and represses IL-6 levels whereas infection by both *S. pyogenes* and BCG show a low TNF-α secretion.
• Information about pro-inflammatory cytokine in response to BCG and *S. pyogenes* infection can be helpful in the understanding of leukocyte migration since they may be bearing same mechanism to evade inflammatory response.

## 3 Data

The infection of epithelial cells is a mandatory step for *Mycobacterium* BCG and *S. pyogenes* dissemination, however the mechanism of such interactions is not completely understood. Secretion of pro-inflammatory cytokines is necessary to construct an appropriate response against an infection.

**Fig. 1:**
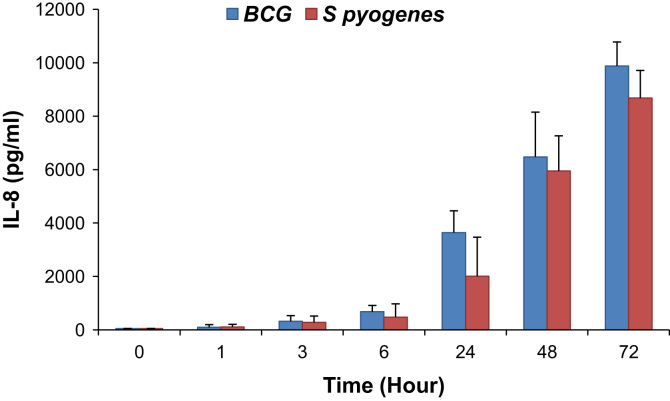
Interleukin-8 levels in media of THP-1 cells infected with BCG and *S. pyogenes* at different time periods post infection.

**Fig. 2:**
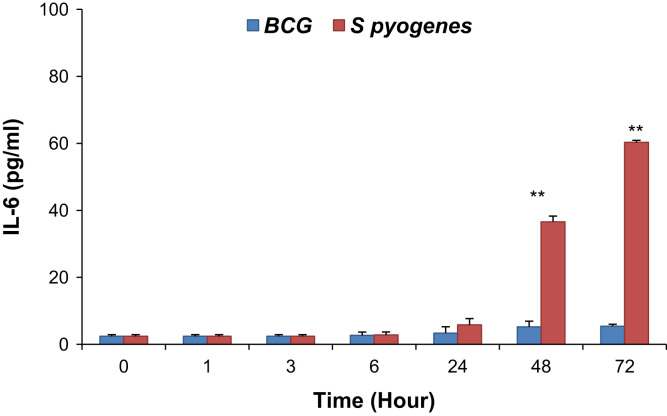
Interleukin-6 levels in media of THP-1 cells infected with BCG and *S. pyogenes* at different time periods post infection. *P* value was calculated by Fisher exact test. **p<0.001.

**Fig. 3:**
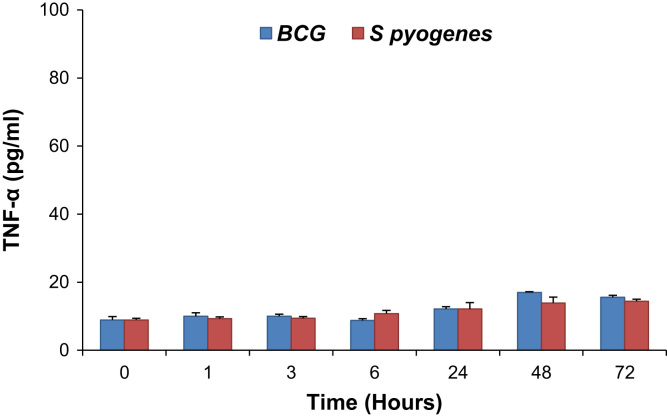
TNF-α levels in media of THP-1 cells infected with BCG and *S. pyogenes* at different time periods post infection.

Induction of inflammatory response by BCG and *S. pyogenes* was estimated as a concentration of the pro-inflammatory cytokines IL-8, IL-6 and TNF-α secreted into media. BCG induced IL-8 response and reached maximum at 72 h post infection while IL-6 was low throughout. *S. pyogenes* induced epithelial secretion of both cytokines IL-8 and IL-6 and reached a maximum level at 72 h post infection (Figs. 1 and 2; **p<0.001) and further declined thereafter (data not shown). However, TNF-α levels were not changed in response to either *S. pyogenes* or BCG (Fig. 3; **p<0.001).

## 4 Experimental design, materials and methods

### 4.1 Bacterial strains and growth conditions

*M. bovis* BCG Danish strain 1331 was grown in Middlebrook 7H9 culture medium, supplemented with 10% Middlebrook ADC Growth Supplement (Sigma-Aldrich, St. Louis, USA) and 50 mg/ml hygromycin (Himedia, India), the culture was dispensed into vials, glycerol was added to a final concentration of 15–25%, and the vials were frozen at −80 °C. Before each experiment, a vial was defrosted, added to 10 ml of 7H9/ADC/hygromycin medium, and incubated with shaking for 72 h at 37 °C [1]. Mycobacteria were then centrifuged for 7 min at 3000 *g*, washed two times with sterile PBS, and re-suspended in 2 ml of sterile PBS. While *S. pyogenes* bacteria were grown to mid-log phase in Todd-Hewitt (TH) broth (Himedia, India), washed and diluted in incubation buffer [2].

### 4.2 Incubation of THP-1 derived macrophage cell line with BCG and S. pyogenes bacteria

THP-1 monocytes were cultured in Roswell׳s Park Memorial Institute medium (RPMI) 1640 (Panbiotech, Germany) supplemented with 10% heat inactivated Fetal Bovine Serum (FBS), HEPES (1%, 1 mM), β-mercaptoethanol (0.1%) with change in medium every third day. The cells were centrifuged at 1000 rpm for 10 min and washed two times with sterile PBS. The numbers of cells were counted on Neubaeur Chamber and 50,000 cells were seeded in each well with 50 nM of PMA (Phorbol 12-myristate 13-acetate; Sigma-Aldrich, St. Louis, USA) for the maturation of the monocytes into macrophage [3].

For the infection experiments, the cells were grown on 12 well plates (50,000 cells/well; Fisher Scientific, UK), infected with BCG (1:1 MOI) and *S. pyogenes* bacteria and than incubated at 37 °C, 5% CO_2_ for three days. Medium alone was used as negative control. Supernatant was collected before infection and at 1, 3, 6, 24, 48 and 72 h post infection for different cytokine profiling.

### 4.3 ELISA

IL-8, IL-6 and TNF-α secretion by the infected THP-1 derived cell line was quantified in the supernatant by enzyme-linked immunosorbent assay (ELISA, eBioscience, San Diego, USA) according to manufacturer’s instructions.
