# Supplementary material for: Kinetics of cytokine profile in response to Mycobacterium bovis BCG and Streptococcus pyogenes activated cells
Source: Data Brief. 2016 Mar 3;7:445–8. doi: 10.1016/j.dib.2016.02.061 (PMC4789312; doi:10.1016/j.dib.2016.02.061)
Supplement: Supplementary file 1 — Supplementary material [file mmc1.pdf]

FACTSHEET

Conflict of Interest

Transparency and objectivity are essential in scientific research and the peer review process.

When an investigator, author, editor, or reviewer has a financial/personal interest or belief that could affect his/her objectivity, or inappropriately influence his/her actions, a potential conflict of interest exists. Such relationships are also known as dual commitments, competing interests, or competing loyalties.[1,2]

The most obvious conflicts of interest are financial relationships such as:
- **Direct:** employment, stock ownership, grants, patents.
- **Indirect:** honoraria, consultancies to sponsoring organizations, mutual fund ownership, paid expert testimony.[2]

Undeclared financial conflicts may seriously undermine the credibility of the journal, the authors, and the science itself.[2] An example might be an investigator who owns stock in a pharmaceutical company that is commissioning the research.

Conflicts can also exist as a result of personal relationships, academic competition, and intellectual passion.[2] An example might be a researcher who has:
- A relative who works at the company whose product the researcher is evaluating.
- A self-serving stake in the research results (e.g. potential promotion/career advancement based on outcomes).
- Personal beliefs that are in direct conflict with the topic he/she is researching.

Not all relationships represent a true conflict of interest–conflicts can be potential or actual.[1,2] Some considerations that should be taken into account include: whether the person's association with the organization interferes with their ability to carry out the research or paper without bias; and whether the relationship, when later revealed, make a reasonable reader feel deceived or misled.[3]

Full disclosure about a relationship that could constitute a conflict–even if the person doesn't believe it affects their judgment–should be reported to the institution's ethics group and to the journal editor to which a paper is submitted. All publishers require disclosure in the form of a cover letter and/or footnote in the manuscript.

A journal may use disclosures as a basis for editorial decisions and may publish them if they are believed to be important to readers in judging the manuscript. Likewise, the journal may decide not to publish on the basis of the declared conflict.

According to the U.S. Office of Research Integrity, having a conflict of interest is not in itself unethical, and there are some that are unavoidable.[1] Full transparency is always the best course of action, and, if in doubt, disclose.

Guide to Conflict of Interest and How to Prevent It*

Action	What is it?	Is it unethical?	What should you do?
**An undisclosed relationship that may pose a conflict of interest**	Neglecting to disclose a relationship with a person or organization that could affect one's objectivity, or inappropriately influence one's actions.	**Yes.**<br><br>Some relationships do not necessarily present a conflict. Participants in the peer-review and publication process must disclose relationships that could be viewed as potential conflicts of interest.[2]	■ When submitting a paper, state explicitly whether potential conflicts do or do not exist.<br>■ Indicate this in the manuscript on a conflict-of-interest notification page, with additional detail. If necessary, include a cover letter with the manuscript.<br>■ Investigators must disclose potential conflicts to study participants and should state in the manuscript whether they have done so.<br>■ Reviewers must also disclose any conflicts that could bias their opinions of the manuscript.[2]
**An undisclosed funding source that may pose a conflict of interest**	Neglecting to disclose the role of the study sponsor(s), if any, in study design; in the collection, analysis, and interpretation of data; in the writing of the report; and in the decision to submit the paper for publication.	**Yes.**<br><br>Undeclared financial conflicts may seriously undermine the credibility of the journal, the authors, and the science itself.[2]	■ When submitting a paper, a declaration (with the heading 'Role of the funding source') should be made in a separate section of the text and placed before the References.<br>■ Describe the role of the study sponsor(s), if any, in study design; in the collection, analysis, and interpretation of data; in the writing of the report; and in the decision to submit the paper for publication.<br>■ Editors may request that authors of a study funded by an agency with a proprietary or financial interest in the outcome sign a statement, such as "I had full access to all of the data in this study and I take complete responsibility for the integrity of the data and the accuracy of the data analysis."[2]

*When in doubt, always consult with your professor, advisor, or someone in a position of authority who can guide you to the right course of action.

References
1. Office of Research Integrity U.S. Department of Health and Human Services. A brief overview on Conflict of Interests. Available at: http://ori.hhs.gov/plagiarism-35. Accessed on September 3, 2012.
2. International Committee of Medical Journal Editors. Uniform Requirements for Manuscripts Submitted to Biomedical Journals: Ethical Considerations in the Conduct and Reporting of Research: Conflicts of Interest. Available at: http://www.icmje.org/ethical_4conflicts.html. Accessed on September 2, 2012.
3. Committee on Publication Ethics (COPE). Committee on Publication Ethics (COPE). Guidelines on Good Publication Practice. 1999. Available at: http://publicationethics.org/static/1999/1999pdf13.pdf. Accessed on September 6, 2012.
